# Atrazine: cytotoxicity, oxidative stress, apoptosis, testicular effects and chemopreventive Interventions

**DOI:** 10.3389/ftox.2023.1246708

**Published:** 2023-10-09

**Authors:** Sunny O. Abarikwu, Ogechukwu E. Ezim, Cynthia N. Ikeji, Ebenezer O. Farombi

**Affiliations:** ^1^ Reproductive Biology and Molecular Toxicology Research Group, Department of Biochemistry, University of Port Harcourt, Choba, Nigeria; ^2^ Drug Metabolism and Toxicology Research Laboratories, Department of Biochemistry, College of Medicine, University of Ibadan, Ibadan, Nigeria

**Keywords:** atrazine, oxidative stress, antioxidant vitamins, flavonoids, cytotoxicity, testes, apoptosis

## Abstract

Atrazine (ATZ) is an environmental pollutant that interferes with several aspects of mammalian cellular processes including germ cell development, immunological, reproductive and neurological functions. At the level of human exposure, ATZ reduces sperm count and contribute to infertility in men. ATZ also induces morphological changes similar to apoptosis and initiates mitochondria-dependent cell death in several experimental models. When *in vitro* experimental models are exposed to ATZ, they are faced with increased levels of reactive oxygen species (ROS), cytotoxicity and decreased growth rate at dosages that may vary with cell types. This results in differing cytotoxic responses that are influenced by the nature of target cells, assay types and concentrations of ATZ. However, oxidative stress could play salient role in the observed cellular and genetic toxicity and apoptosis-like effects which could be abrogated by antioxidant vitamins and flavonoids, including vitamin E, quercetin, kolaviron, myricetin and bioactive extractives with antioxidant effects. This review focuses on the differential responses of cell types to ATZ toxicity, testicular effects of ATZ in both *in vitro* and *in vivo* models and chemopreventive strategies, so as to highlight the current state of the art on the toxicological outcomes of ATZ exposure in several experimental model systems.

## 1 Introduction

Atrazine (ATZ; 2-chloro-4-ethylamino-6-isopropylamino-s-triazine) is a triazine herbicide that is used to control the growth of broadleaf and grassy weeds. Atrazine is commonly spread pre-emergence to maize, coffee, sorghum, sugarcane, wheat, in conifer forests, on golf courses, on Christmas tree farms and household lawns. Currently, ATZ and related triazines are one the most extensively utilized agricultural herbicides in the world and the most frequently patronized herbicide in Nigeria (Adesina et al., 2014), and with an annual production of over 36,000 tonnes (Jablonowski et al., 2011; Chang et al., 2022). The extensive use of ATZ has given rise to its detection in the environment, largely in surface and groundwater (Goldman, 1994; Owagboriaye et al., 2022) and in maize plants (Santos-Hernández et al., 2018; Yu et al., 2021). ATZ at a concentration of 0.2 ppb has been detected in 2–3 million people who use groundwater as their main source of drinking water (Goldman, 1994), as such ATZ has been given a sizeable deal of scientific audit by both governmental and scholarly researchers. ATZ can be found in the environment at concentrations as substantial as 21 ppb in groundwater, 42 ppb in surface waters, 102 ppb in water basins in farm areas, and up to 224 ppb in streams (Kolpin et al., 1998; Owagboriaye et al., 2022; Roh et al., 2023). The U.S. Environmental Protection Agency (EPA) has set the maximal limit of ATZ allowed in drinking water (MCL) at 3 ppb and has designated ATZ as a Restricted Use Pesticide in an attempt to minimize human exposure to ATZ through drinking contaminated groundwater (Costa Silva et al., 2010; Powell et al., 2011). However, in occupational set-up, humans are exposed to ATZ at a thousand-fold higher concentration than seen in residential exposure. Because triazine herbicides are dominantly applied to the maize cropping fields, the European legislation has set the maximum residue level for ATZ in corn as 0.25 mg kg^−1^ (Elvis et al., 2015; Yu et al., 2021). In fact, ATZ is thought to be highly selective for maize, since their roots could efficiently metabolize ATZ to OH-atrazine; and the formation of glutathione conjugates in the aerial parts of the plant (Tasli et al., 1996). Evidence from epidemiological reports have also indicated a plausible connection between exposure to ATZ-containing agrochemicals in workers handling the herbicide and an upsurge of diverse neoplastic diseases (Schroeder et al., 2001; Hopenhayn-Rich et al., 2002; Alavanja et al., 2003). Because of its unacceptable levels on groundwater, ATZ use was restricted in the European Union in 2004 (Zeljezic et al., 2006; Ackerman, 2007). However, ATZ-containing formulations have continued to be produced for export in some European member states (United Kingdom, Spain, Portugal and Ireland). In Africa and other developing countries where there are weak policies on agrochemicals usage and disposal, the contamination of drinking waters might even be higher. As a result of this and the constancy of ATZ in groundwater, segments of the European and African populations will still be brought into contact with this active ingredient. Although the strict ranking of ATZ as an endocrine disrupting compound has been discussed, a lot of sequelae on living organisms have been suggested (Solomon, 2009).

The endocrine disrupting effects of ATZ in birds, reptiles, fish, amphibians and mammals (Bisson and Hontela, 2002; Hayes et al., 2003; Fan et al., 2008; Zaya et al., 2011; Abarikwu et al., 2021); immunotoxic properties (Brodkin et al., 2007; Thompson et al., 2015) and effects on sperm qualities in animals at low exposure levels have been reported (Saalfeld et al., 2018). At higher exposure levels, ATZ causes a number of effects that are similar across several animal species, e.g., developmental delays and abnormalities (Tavera-Mendoza et al., 2002; Nieves-Puigdoller et al., 2007), steroidogenesis and spermatogenesis abnormality, induction of oxidative stress and cytotoxicity and apoptosis (Abarikwu et al., 2011a, 2012a; Victor-Costa et al., 2010; Pogrmic et al., 2009; Pogrmic-Majkic et al., 2010). Considering the relevance of genotoxic and oxidative damage in the harmful effects of several environmental and biological toxic chemicals such as agrochemicals, there has been no systematic study on the cytotoxicity, apoptosis and oxidative stress effects of ATZ in animal and cellular model systems. It is consequently imperative to point-out molecular variables of oxidative stress that detect effects of ATZ in both *in vivo* and *in vitro* experimental model systems. We present a review about ATZ effects, focusing on some target *in vivo* and cell-based *in vitro* systems in order to achieve a better grasp of its cellular mechanism of actions.

### 1.1 The Cytotoxicity of ATZ. data from *In Vitro* Study

The cytotoxic and DNA damaging effects of ATZ have been tested and confirmed in a variety of cell types; although most of the studies present inconsistent and conflicting results (Pino et al., 1988; Clements et al., 1997; Ribas et al., 1998; Taets et al., 1998). For instance, three cytotoxicity techniques: sister-chromatid exchanges, chromosome aberrations and micronuclei assays suggested that ATZ (5–100 μg/mL; 23.2–462 µM) lack the capacity to induce clastogenic and aneugenic damages in cultured human lymphocytes (Ribas et al., 1998). Other *in vitro* studies demonstrated that cultured human lymphocytes exposed to triazines (ATZ, simazine, and cyanazine) did not induce sister-chromatid exchanges or chromosome aberrations up to the limits of the doses of the chemicals that were severely toxic (Kligerman et al., 2000a). The chemical was also found not to be toxic to sheep peripheral blood phagocytes and lymphocytes even up to 100 mM, whereas in some studies, even at concentrations of ATZ as low as 0.0001 μg/mL (0.0005 µM), DNA damage were demonstrated in human lymphocytes cultures as measured by comet assay and chromosomal aberration analysis (Meisner et al., 1992). Similar results were also found when chromosome aberrations, sister chromatid exchanges and mitotic index, were applied in human peripheral lymphocytes exposed *in vitro* to ATZ at concentrations between 5 and 51 µM. Interestingly, the dose-related increase in the percent of aberrant cells and of sister chromatid exchanges were dependent on ATZ induced cytotoxicity as measured by Trypan blue exclusion assay (Lioi et al., 1998). At the highest concentration of ATZ exposed to the cultured human lymphocytes, glucose 6-phosphate dehydrogenase activity, either remained stable or decreased, when the cytotoxicity effect was increased (Lioi et al., 1998). This observation allowed the speculation that the increased sister-chromatid exchanges and chromosome aberrations after ATZ exposure, accompanied by a change in the cell redox state, confirms that reactive oxygen intermediates are involve in the cytotoxicity effect of ATZ in cultured lymphocytes. Thus, the thought that the genetic toxicity of ATZ and triazine chemicals could alter reactive oxygen intermediates in mammalian cells through a mechanism that involves glucose 6-phosphate dehydrogenase was demonstrated (Lioi et al., 1998). Hence, the initial response of the cells to ATZ would involve depleting of intracellular GSH pools, and subsequently, the inactivation of glucose 6-phosphate dehydrogenase activity/expression. It is therefore rationale to assume that when the exposure of cultured human lymphocytes to ATZ is sustained, the efficiency of the hexose monophosphate shunt to replenish the GSH pools is hampered with, together with it capacity to protect human lymphocytes against oxidant injury (Lioi et al., 1998). Furthermore, comet assay applied on erythrocytes isolated from the tadpoles, *Rana catesbeiana* exposed to ATZ showed DNA damage that appears to be dependent on ATZ dosages (Clements et al., 1997). Other studies have shown that these genotoxic effects are minimal, even if it exists. Roloff et al. (1992) reported little chromosome damage in human lymphocytes exposed to 0.005 µM (0.001 μg/mL) ATZ concentrations. ATZ did not also induce genotoxic damage by comet and DNA diffusion assay in human lymphocytes at concentrations between 0.047 and 4.7 μg/mL, whereas the commercial preparations of ATZ containing adjuvant mixtures could increase DNA damage in lymphocytes (Zeljezic et al., 2006), suggesting that the commercial formulations contain other substances that represent a genotoxic risk to human lymphocytes. Although the DNA damaging effects for non-cytotoxic doses of ATZ appear controversial (Meisner et al., 1992), higher concentrations would be more consistent with the genotoxicity of ATZ. However, sister chromatid exchanges analysis at metaphase cells are not appropriate techniques to use for this endpoint, since affected cells are delayed in G2-phase and do not proceed to mitosis (Malik et al., 2004). Furthermore, as the cytotoxicity studies for high concentrations of ATZ did not also demonstrate any increase in homologous recombinational events (Malik et al., 2004), a genotoxic mode of action of ATZ cannot be assumed in cultured human lymphocytes. Therefore, the variations in the sister chromatid exchanges analysis after exposures to certain concentrations of ATZ are due to polarities in cell cycle kinetics of cultured lymphocytes, rather than to a true biological disparity in the cytogenetic marker used (Malik et al., 2004). Hence, the general notion for most studies on the genotoxicity of ATZ in human cultured lymphocytes is to report negative results.

The cytoxicity of ATZ has also been demonstrated in gonadal experimental model systems. Clastogenicity was reported in the Chinese Hamster ovary (CHO) cells when flow cytometry was the cytotoxicity technique applied to determine the chromosomal damaging potentials of ATZ at the US EPA maximum contamination level of 3 ppb (0.0139 µM), and the highest contamination level of 18 ppb (0.0835 µM) found in a community drinking water supplies (Taets et al., 1998). Another study using the CHO cells found that ATZ at 20 μg/mL (20,000 ppb) caused a 19% growth decrease after 72 h and at 80 μg/mL (80,000 ppb), the cell viability was inhibited by 55% (Kmetic et al., 2008). The rates of porcine oocyte maturation and quality were reduced after exposure *in vitro* to 200 μM ATZ. When 5-bromo-deoxyuridine assay was applied to test the cell proliferative capacity, the population of proliferating cells was found to decrease. The cytotoxicity of ATZ effect also resulted in the disruption of the spindle morphology, maturation-promoting factor activity; mitochondrial membrane potential and DNA damage response as shown by TUNEL assay (Yuan et al., 2017). High levels of superoxide radicals, increase cathepsin B activity and the decrease in GSH concentration was assumed to be responsible for the impaired developmental competence of porcine oocyte (Yuan et al., 2017). The viability of testicular cells (Leydig and Sertoli-germ cells) that were treated with 232 μM (50 μg/mL) ATZ concentration and subjected to the 2,5-diphenyl-2H-tetrazolium bromide salt (MTT) assay assays were found to be decreased (Abarikwu et al., 2011a; Abarikwu et al., 2012). The neutral red uptake (NRU) assay which allows viable cells to incorporate and bind the supravital dye neutral red in the lysosomes, also showed similar cytotoxicity pattern in Leydig cells but were less sensitive than the MTT assays (Abarikwu et al., 2011a). This concentration of ATZ that was found to be cytotoxic in testicular cells was also required to decrease the viability of the rat pheochromocytoma (PC12) cells (Abarikwu et al., 2011b). In this study on ATZ model of neuronal injury, the four cytotoxicity techniques: MTT, NRU, lactate dehydrogenase leakage and trypan blue assays confirmed the cytotoxic damage of ATZ in the PC12 cells. If the 24 h required for the manifestation of the cytotoxic response (Abarikwu et al., 2011b) is compared to the 48 h for the MTT assay and 72 h for the NRU assay; required for the onset of cell death in testicular cells (Leydig cells) (Abarikwu et al., 2011a), it could suggest that neuronal cell lines could be more vulnerable to the cytotoxicity of ATZ than testicular cells. Additionally, ATZ concentration (300 μM, 65 μg/mL) slightly higher than those reported in the above studies (Abarikwu et al., 2011a; Abarikwu et al., 2011b; Abarikwu et al., 2012) was also observed to decrease the proliferation and cellular expansion of the human neuroblastoma (SH-SY5Y) cells (Abarikwu et al., 2011c). This was also detected in cell viability studies as demonstrated by the MTT and NRU assays and DNA ladder-like formation detected when ethidium bromide stained agarose gel electrophoresis was applied to check the mechanism of the cell death (Abarikwu et al., 2011c).

The concentration-dependent cytotoxicity of ATZ has also been demonstrated across other mammalian cell types and fish cells. Tchounwou et al. (2001) found that ATZ concentration at 100 μg/mL (100, 000 ppb) is non-toxic to the human hepatoma cell line, HepG2. The chemical was also not toxic to rat primary hepatocytes (Sawicki et al., 1998). We had previously reported that ATZ at concentration of 300 μM (65 μg/mL); a concentration lower than that used in Tchounwou study was able to inhibit growth of human neuronal cell line (SH-SY5Y cell line) (Abarikwu et al., 2011c). An ATZ concentration of 625 ppb or higher was needed to cause a decrease in HepG2 cell proliferation compared to control cells (Powell et al., 2011). Interestingly, concentrations of ATZ lower than 625 ppb were not sufficient to inhibit growth of immortalized HepG2 cells (Powell et al., 2011). However, in a previous study, ATZ at a concentration as small as 0.8 ppb was sufficient to decrease the growth of normal human fibroblast cells (Dhanwada et al., 2003). This value is 3.2 times less than the MCL of 3 ppb fixed by the EPA and is frequently detected in drinking water supplies (Gely-Pernot, 2017). Furthermore, the 625 ppb value is 3.5–10 times smaller than the concentration reported in studies where immortalized cells had been used (Sanderson et al., 2000; Laville et al., 2006; Rowe et al., 2007) and 160 times smaller than was reported in HepG2 cells (Tchounwou et al., 2001). The cytotoxic effects of ATZ was also reported by the MTT assay in cultured grass carp (*Ctenopharyngodon idellus* cell line, ZC7901), with IC_50_ value ranging from 11.6 ± 6 0.5 mg/L to 199.0 ± 6 7.8 mg/L (Liu et al., 2006), indicating substantial flexibility in the cytotoxic responses of fish cells to ATZ exposure. Moreover, DNA fragmentation was detected by the TUNEL reaction and agarose gel electrophoresis in dose-and time-dependent fashion. The application of the MTT technique on this experimental model system, confirmed the cytotoxicity of low concentration of ATZ (9.4–47.2 mg/L) in fish cells.

The cytotoxicity of ATZ was also tested at doses ranging from 10–500 μM in human embryonic stem cells (hESC), a differentiation model system that is used to test the toxicity of chemicals at divergent phases of neural differentiation *in vitro.* In this study, hESC were differentiated into neural stem cells before they were terminally differentiated to neurons and glial cells after 21 days. The application of the cell counting kit-8 (CCK-8) cell viability assay showed that ATZ could inhibit hESC viability and proliferation, and the numbers of colonies were dose-dependently decreased reaching almost 50% at 200 μM dose of ATZ after a long period of exposure (Shan et al., 2021).Other studies demonstrated that exposure to low-dose ATZ ranging from 0.0014–0.14 μM (0.3–30 ppb) for 4 days prior to differentiation and completion of differentiation, can result in long-lasting changes in epigenome and increase risks of synuclein alpha (SNCA)-related Parkinson’s disease in a pre-differentiation SH-SY5Y model system (Xie et al., 2021). The cytotoxicity observed in this study was minimal at the tested ATZ concentrations when tested by the MTT assay, suggesting that neuronal differentiation was affected without changes in the viability of cells (Xie et al., 2021). The effects of ATZ (12–300 μM) exposure on N27 rat dopaminergic cells, also confirmed that ATZ could alter the morphology of undifferentiated N27 cells, because after 48 h of exposure, differentiating N27 cells exhibited increased numbers of neurites and longer neurite length (Lin et al., 2013) similar to the altered neurite outgrowth reported at pre-differentiation exposure of SH-SY5Y to ATZ (Xie et al., 2021). It is interesting to also note that the sexual differentiation in amphibians also suffers dramatic changes at doses as low as 0.1 μg/L of ATZ (Hayes et al., 2002). It is therefore reasonable to assume that developmental exposure to low dose ATZ has the potential to cause long-lasting neurological variations (neural toxicity) and reproductive phenotypic changes in animal models. This is interesting to know, and has important environmental and public health concern, because the low dose 3 ppb and high dose 30 ppb ATZ concentrations employed in these studies are the present EPA regulation standard of ATZ in drinking waters, and the minimal dose of ATZ that can elicit gene expression changes without cytotoxicity effects, that are considered safe in drinking water or safe for limited human exposure, respectively (Rohr and McCoy, 2010; Xie et al., 2021).

Similarly, conflicting results on the cytotoxicity of ATZ in intestinal cells have been demonstrated. For instance, ATZ concentrations ranging from 1 to 10 μM (215.7–2157 ppb) increased the proliferation of human intestinal epithelial cells after 72 h, a mechanism associated with cancer development (Green and Reed, 1998).The application of trypan blue exclusion assay for cellular viability and the MTT assay for cell growth study on the normal rat IEC-6 intestinal and human colonic epithelial cell cultures produced a growth-stimulatory effect of ATZ, and the lowest dose (0.5–10 µM) was even more potent than higher doses (50 µM) in stimulating cell growth (Green and Reed, 1998). Because the growth and viability of normal rat intestinal cells and human colonic epithelial cell cultures treated with the DMSO vehicle were not influenced, lead to the suggestion that the growth rate increases in ATZ treated rat intestinal and colonic epithelial cell cultures are not due to the vehicle control (DMSO). However, the human colonic epithelial cell cultures are more sensitive to the effects of ATZ in sustaining cell growth than the rat intestinal cell cultures (Green and Reed, 1998). At concentration of 50 µM ATZ and above, ATZ was found to provoke substantial cytotoxic effects on the human intestinal Caco-2 cells. This brings about the decrease in cell proliferation rate and viability (Olejnik et al., 2010). Moreover, long-term exposure of the intestinal cells to ATZ at non-cytotoxic doses (1–10 μM) inhibited cell maturation and decline the transepithelial electrical resistance (Olejnik et al., 2010). The MTT test and the Trypan blue exclusion assay verified the cytotoxicity of ATZ, as both a dose- and time-dependent event in Caco-2 cells cultures (Olejnik et al., 2010). Thus, unlike the normal rat intestinal and human colonic cells, the human intestinal Caco-2 cells are more sensitive to the cytotoxic effect of ATZ (Olejnik et al., 2010). The reasons for these contrasting results are, apart the cancerous nature of Caco-2 cells, it is thought that ATZ causes loss of growth control in normal intestinal cells leading to its fast proliferation which allows the propagation of the mutated cells and ultimately leads to neoplasia (Olejnik et al., 2010). Furthermore, the growth of colonic epithelial cells may be responsive to the estrogenic activity of ATZ, such that the higher the number of estrogen receptors in normal cells, more than those of transformed cells, the higher the capacity of ATZ to stimulate normal intestinal cell proliferations (Olejnik et al., 2010). Additionally, DNA breaks and alkali labile lesions were found in gastric mucosa cells, when DNA alkaline elution technique was applied as the genotoxicity assay (Pino et al., 1988).

It is interesting to speculate that some aromatase sensitive cell types appear to exhibit consistency when they are tested for ATZ cytotoxicity. Laville et al. (2006) reported that ATZ at 10 μM (2157 ppb) did not alter the viability of the human choriocarcinoma JEG-3 cell line after 24 h as verified by the MTT assay; although this concentration could induce the activity of the aromatase enzyme, a very important molecular target of triazine herbicides, and increase or inhibit the viability of other cell types not expressing aromatase (Olejnik et al., 2010; Green and Reed, 1998). Several herbicides with endocrine disrupting effects like ATZ could also induce or inhibit aromatase activity at concentrations even lower than those unable to alter cell viability, suggesting that cytotoxicity and steroidogenic effects share dissimilar molecular events in aromatase responsive cell types (Laville et al., 2006). Another possibility on the unaltered viability of the JEG-3 cells would be that the high expression of aromatase in JEG-3 cells might be protective against cytotoxic responses by ATZ. This appears rationale to assume because human adrenocortical (H295R) and human breast cancer (MCF-7) cell lines which also express high level of aromatase (Sanderson et al., 2001) did not experience cytotoxicity at the same dose applied in the JEG-3 cells (Sanderson et al., 2000). In the work of Sanderson and colleagues, ATZ at concentrations of 0.3–30 μM (64.7–6,471 ppb) did not alter the growth of the H295R cells, even at the highest ATZ concentration tested, and despite the fact that these concentrations caused a dose-dependent increase in aromatase activity (Sanderson et al., 2000) and drastically inhibit the viability of other cell types (Abarikwu et al., 2011c; Powell et al., 2011). These molecular features could be responsible for dampening ATZ cytotoxicity on these cell types.

ATZ cytotoxicity has also been reported in immune cells. For instance, natural-killer cell-specific activity of peripheral blood lymphocytes declined 24 h after ATZ exposure at concentrations of 3–30 μM (647.1–6,471 ppb), however, cell viability was unchanged even at the highest tested concentration of the herbicide (Rowe et al., 2007). CD4^+^ T cells were activated by 30 µM ATZ after 4 days of exposure (Thueson et al., 2015). The ATZ exposure decreased the antigen-driven build-up of CD4^+^ cells, an observation that is at variance with the unaltered numbers of CD4^+^ T cells that was reported earlier in the work of Filipov et al. (2005). This discrepancy was explained by the fact that *in vivo* ATZ exposure was not accompanied with lymphocyte activation. This is because in the absence of the triggering of T cell receptors (TCR), ATZ has only minimal effect on CD4^+^ T cell numbers (Shan et al., 2021). This is clinically relevant because the level of ATZ that produced these effects is lower than their urinary concentrations in high-risk people including farmers (Perry et al., 2001) and is similar or smaller than the levels reported in in vitro studies examining the immunotoxicity of ATZ (Devos et al., 2003; Pinchuk et al., 2007). Interestingly, 30 µM ATZ when applied *in vitro* to the CD4^+^ T cells was not cytotoxic since the frequency of apoptotic or necrotic cells was not raised. However, ATZ completely inhibited CD4^+^ T cell proliferation in a manner that was correlated with the inhibition of T cell activation, suggesting that people that are chronically exposed to ATZ occupationally or in their drinking water may have an altered immune status (Thueson et al., 2015). Recently, Galbiati et al. (2021) speculated that ATZ act on dopamine receptors expressed in immune cells during inflammatory responses for host defense, and alter inflammation-related diseases. Therefore, triazines immunotoxicology remains an important area to explore as it would influence our understanding of the connection between the immune and nervous systems in triazines disease model, e.g., inflammation-related diseases (Thompson et al., 2015). Dendritic cell viability studies with trypan blue exclusion assay showed that ATZ, at concentrations of up to 100 µM was not cytotoxic to bone marrow-derived immature dendritic cell lines (JAWSII DC) after 24 h. The application of annexin-V apoptosis assay to detect apoptosis found that at concentrations of ATZ up to 200 μM, the number of JAWSII cells undergoing apoptosis was increased, whereas at the highest concentration (300 µM), it increased modestly, the percentage of annexin-V positive cells. The phenotypic changes, including loss of surface major histocompatibility complex −1 (MHC-I) was observed at 1 µM concentration of ATZ. In these studies, the proportions of mature dendritic cells were decreased, suggesting that ATZ inhibited dendritic cell maturation even at doses that are regarded to as not cytotoxic (Pinchuk et al., 2007). Furthermore, ATZ-induced degranulation of mast cells was determined by measuring the release of granule-associated β-hexosaminidase from RBL-2H3 cells in which ATZ between the range of 10 nM and 1 μM showed induced rapid degranulation in mast cells. Interestingly, ATZ did not cause any cytotoxic effects on the cells, as evaluated by the trypan blue exclusion assay (Mizota and Ueda, 2006). It may appear that the doses of ATZ that interfered with major biological functions do not alter the viability of immune cells.

The cytotoxicity of ATZ has been tested with other triazines in four human breast cell lines: MCF-7, MDA-MB-231 and MCF-10A. One common feature of these cells is their capacity to respond to endocrine toxicity (Rich and Gabriel, 2012). These breast cancer cells were found to be responsive to endocrine-disruptors, but the triazines (atrazine, cyanazine and simazine) failed to dramatically alter cell densities (Rich and Gabriel, 2012). Although there was a sign of plausible increase with ATZ at 10 nM and cyanazine at 10,000 nM in the MCF-7 cells, a tendency for raised cell viability was observed at 1,000 nM with simazine in MCF-7 and MDA-MB-231 cells, and with no changes for the MCF-10A cells with any of the tested triazines. It appears that the existence of the estrogen receptors in these cells enhances the stimulatory effects of these chemicals and inflates cell growth, yet when these receptors are missing, the chemicals may be initiating cell death through apoptotic pathways (Rich and Gabriel, 2012). The viability of MDA-MB-231 cells, an estrogen independent breast cancer cell and the non-cancerous MCF-10A breast cells were not altered much after exposure to ATZ, even though it shows a trend to decrease except for simazine which dramatically increased the cell viability, suggesting that this triazine unlike ATZ responded diversely in the cells and/or that different tumors respond differently to triazine chemicals, a scenario that could influence the likely stimulatory and cytotoxicity actions of these chemicals (Rich and Gabriel, 2012). This variance in the responsiveness of different cells to ATZ toxicity are thought to be due to several points such as the choice of solvent (e.g., dimethyl sulfoxide or ethanol) used to dissolve the ATZ (Powell et al., 2011) and the ATZ estrogenic activity (Olejnik et al., 2010) as previously mentioned above. It is known that cultures of non-transformed cells are more estrogen-sensitive than the tumor cells (Potter, 1995). To affirm this speculation, Manske et al. (2004) reported that when DMSO was used as a solvent for ATZ, a 12.5-fold higher level of ATZ (10 ppb) was required to inhibit growth of normal human fibroblasts. Also, the proliferation of the estrogen-sensitive MCF7-BUS human breast cancer cells was not affected by ATZ concentration of up to 10 μM (Oh et al., 2003). It seems that ATZ can inhibit proliferation, viability and induces DNA damage in different cell types under *in vitro* settings, mostly at levels that do not frequently occur in the environment (Table 1). Furthermore, low-level of ATZ at concentrations that are environmentally material can lead to inhibition in the growth of normal cells raising the possibility that normal cells are more susceptible to the cytotoxicity of ATZ than immortalized cells. From all the data above, and within the context of ATZ model of cellular toxicity, cytotoxicity occurs at ATZ concentrations different from the doses that interferes with other important biologic functions, e.g., enzyme activity and/or expression, and that primary cells are more sensitive than cell lines to ATZ cytotoxic effect, which might also be affected by several factors including cell types (estrogen-responsive) and choice of the solvent used to dissolve the ATZ.

**TABLE 1:** Cytotoxicity effects of different concentrations of atrazine (μM) in different cell types.

Concentrations (μM)	Cell Types	Effects on cell viability	References
4 × 10^−3^	Human fibroblasts	Decreased	Dhanwada et al. (2003)
10	Caco-2	No change	Green and Reed (1998)
30	H295R	No change	Sanderson et al. (2000)
30	Peripheral blood lymphocytes	No change	Rowe et al. (2007)
2.9–23.2	HepG2	Decreased	Powell et al. (2011)
50–1,000	Rat hepatocytes	No change	Sawicki et al. (1998)
200–300	Mouse dendritic cells	Decreased	Pinchuk et al. (2007)
232	Rats testicular cells	Decreased	Abarikwu et al. (2011a)
232	PC12	Decreased	Abarikwu et al. (2011b)
250	Caco-2	Decreased	Olejnik et al. (2010)
300	SH-SY5Y	Decreased	Abarikwu et al. (2011c)
93–371	CHO-K1	Decreased	Kmetic et al. (2008)
463	HepG2	No change	Tchounwou et al. (2001)
10–500	hESC	Decreased	Shan et al. (2021)
30	CD4^+^ T	No change	Thueson et al. (2015)
0.01–10	Splenic lymphocytes	No change	Chen et al. (2015)

### 1.2 Apoptosis-inducing effects of ATZ

Atrazine-induced apoptosis has been investigated in several experimental model systems (Table 2). A study developed by Liu et al. (2006) demonstrated that cells that are incubated with ATZ-presented a series of structural changes, including shrinking of the nucleus, flanking of chromatin to the shape of thick granular caps, and generation of apoptotic bodies. Moreover, TUNEL assay and agarose gel electrophoresis were used to detect the fragmented DNA. This was the first proof that ATZ could cause apoptosis *in vitro*. Exposure to agrochemicals, e.g., ATZ is assume to be one of the stressful cellular situations that could trigger some molecular events, e.g., expression of p53 protein. This protein gauges DNA damage and function as a transcription factor controlling genes, which regulate cell growth, apoptosis and DNA repair (Cozmei et al., 2002). p53 expression was found to be increased in peripheral lymphocytes of rats chronically treated with ATZ (Cantemir et al., 1997). The peripheral mononuclear cells of humans occupationally exposed to ATZ also showed increased expression of p53 (Cozmei et al., 2002). Tian et al. (2018) observed that the proliferation of H22 cells after been treated with 0.01 and 0.1 µM ATZ increased in the absence of changes in the percentage of apoptotic cells. Additionally, the tumor size and mass of ascites were remarkably expanded in an orthotopically implanted hepatocarcinoma tumor C57BL/6 mice model. p53 expression was downregulated, whereas those of cyclin-D1, MMP2, Stat3, VEGF and C-myc was upregulated by ATZ, suggesting that ATZ activated Stat3 signaling and induced the proliferation and seizure of hepatocellular carcinoma cells. The H22 cells that were exposed to ATZ at 0.01 μM displayed an increase in proliferation much more than those exposed to 0.1 μM ATZ when tested by the MTT proliferation assay. The increase in H22 cells proliferation was also accompanied with a decline in the number of cells in the G2 phase and a rise in the populations of cells in the S phase, suggesting that ATZ induces the accumulation of cells in the S phase and moves the H22 cells quickly through the G2 phase (Tian et al. (2018). The numbers of apoptotic cells in human lymphocytes have also been reported to escalate when treated with commercial preparations containing ATZ (Zeljezic et al., 2006). The apoptotic nuclei of lymphocytes going through apoptosis or necrosis were distinct from normal cells in having a foggy or vague outline without exact margins because of the nucleosomal-sized DNA dispersing into the agarose. Necrotic cell nuclei are larger and are poorly delineated. They have a distinct, defined outward margin of the DNA halo and a proportionate homogenous halo appearance. Conversely, cells that are not necrotic or apoptotic but have damaged DNA have well-defined nuclei with a broad halo and defined external boundary.

**TABLE 2:** Expressions of apoptosis-related genes in differerent cell types exposed atrazine.

Concentrations (μM)	Cell types	Expressions of genes	Genes	Reference
300	SH-SY5Y	Decreased	p53	Abarikwu et al. (2011c)
		Decreased	p21	
		Decreased	Bax	
		Decreased	Bcl-2	
232	PC12	Increased	p53	Abarikwu et al. (2011b)
		Increased	Bax	
		Increased	Caspase-3	
		Increased	Caspase-9	
		Decreased	Bcl-2	
0.23–2.32	HepG2	Decreased	Cyclin B	Powell et al. (2011)
		Decreased	Cyclin E	
0.01 and 0.1	H22	Decreased	p53	Tian et al. (2018)
		Increased	Cyclin-D1	
		Increased	VEGF	
		Increased	C-myc	
10		Increased	Caspase-3	Ge et al. (2021)
	Lymphocytes	Increased	Caspase-9	
		Decreased	Bcl-2	
1–30	Human placenta	Decreased	p53	Ibrahim et al. (2015)
		Increased	Bcl-2	

Shan et al. (2021) treated hESC and neural stem cells to ATZ for up to 24 h and observed that ATZ caused dissimilar toxic susceptibility on these cells. For instance, ATZ stopped the G1 phase of neural stem cells *by* down-regulating the cyclin-dependent kinase 4 and 2 (CDK4 and CDK2), which occluded more cells to traverse the G1/S phase nodes and inhibited the mitosis of neural stem cells. Thus, considering the neurotoxicity of ATZ, it is believed that ATZ not only target the dopaminergic system but also the glutamatergic neurons and astrocytes (Filipov et al., 2007; Shan et al., 2021). In addition, MKI67 (marker of proliferation Ki-67) and PCNA (proliferating cell nuclear antigen) gene expressions along with CCND1 which moves cells from G1 into S phase were decreased, but the percentages of early apoptosis and late apoptotic cells showed no obvious changes at the tested doses (100–500 μM) of ATZ treatment (Shan et al. (2021). The recent findings by Galbiati and colleagues on the connection between the immune and nervous systems in endocrine disruption of ATZ, confirms the relevance of the dopaminergic system in the neurotoxicity of triazines (Galbiati et al., 2021). Manske et al. (2004) did not detect any increase in apoptosis in human fibroblast exposed to low doses of industrial grade ATZ. However, in the study of Greenlee et al. (2004) herbicide preparations containing ATZ suitable for commercial purposes increased the rate of apoptosis in exposed murine embryos.

**FIGURE 1:**
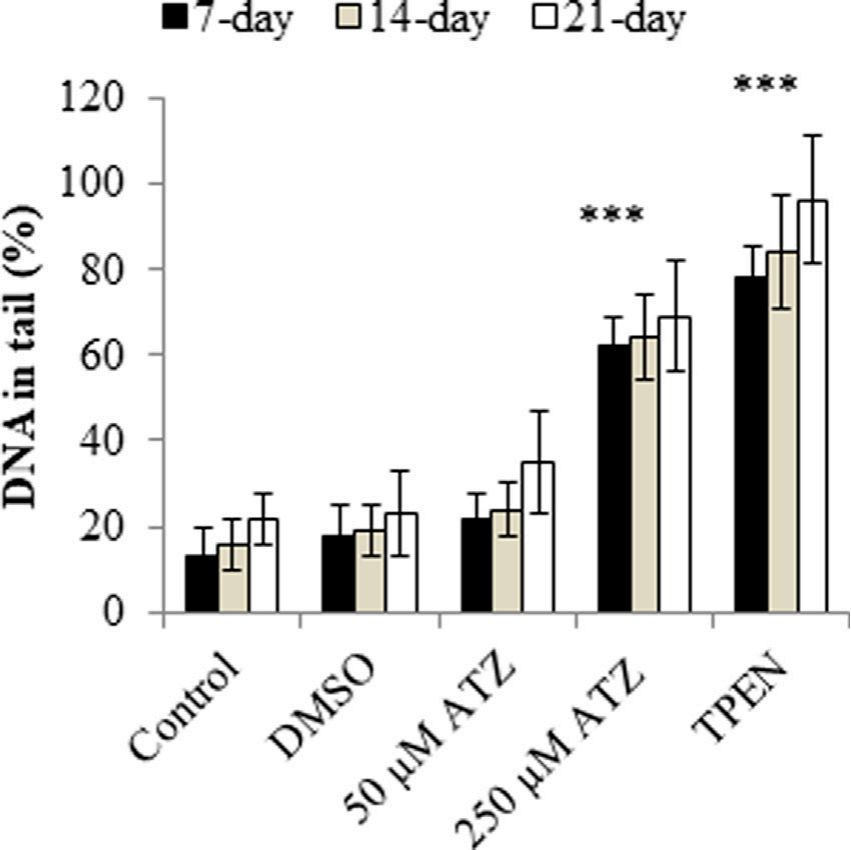
DNA damage in 7-, 14-, and 21-day-old Caco-2 cells treated with 50 and 250 μM ATZ for 24 h. DNA damage is expressed as the percentage of DNA in the comet tail. (***p < 0.001). Source: Redrawn from data of Olejnik et al. (2010).

**FIGURE 2:**
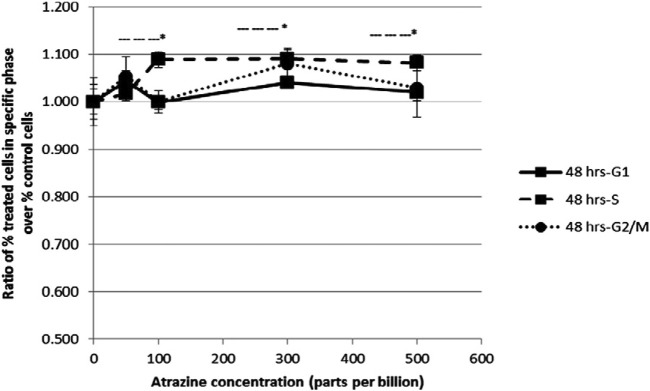
Flow cytometric analysis of HepG2 cells after a 48 h exposure to increasing concentrations of atrazine. Cells were harvested and DNA was stained with propidium iodide for flow cytometry analysis. The ratio of the percent of treated cells in a specific phase of the cell cycle (G1, S or G2/M) over the percent of untreated control cells in the same phase was determined. The ratio ±standard error of the mean (SEM) is indicated. Source: Powell et al., 2011.

**FIGURE 3:**
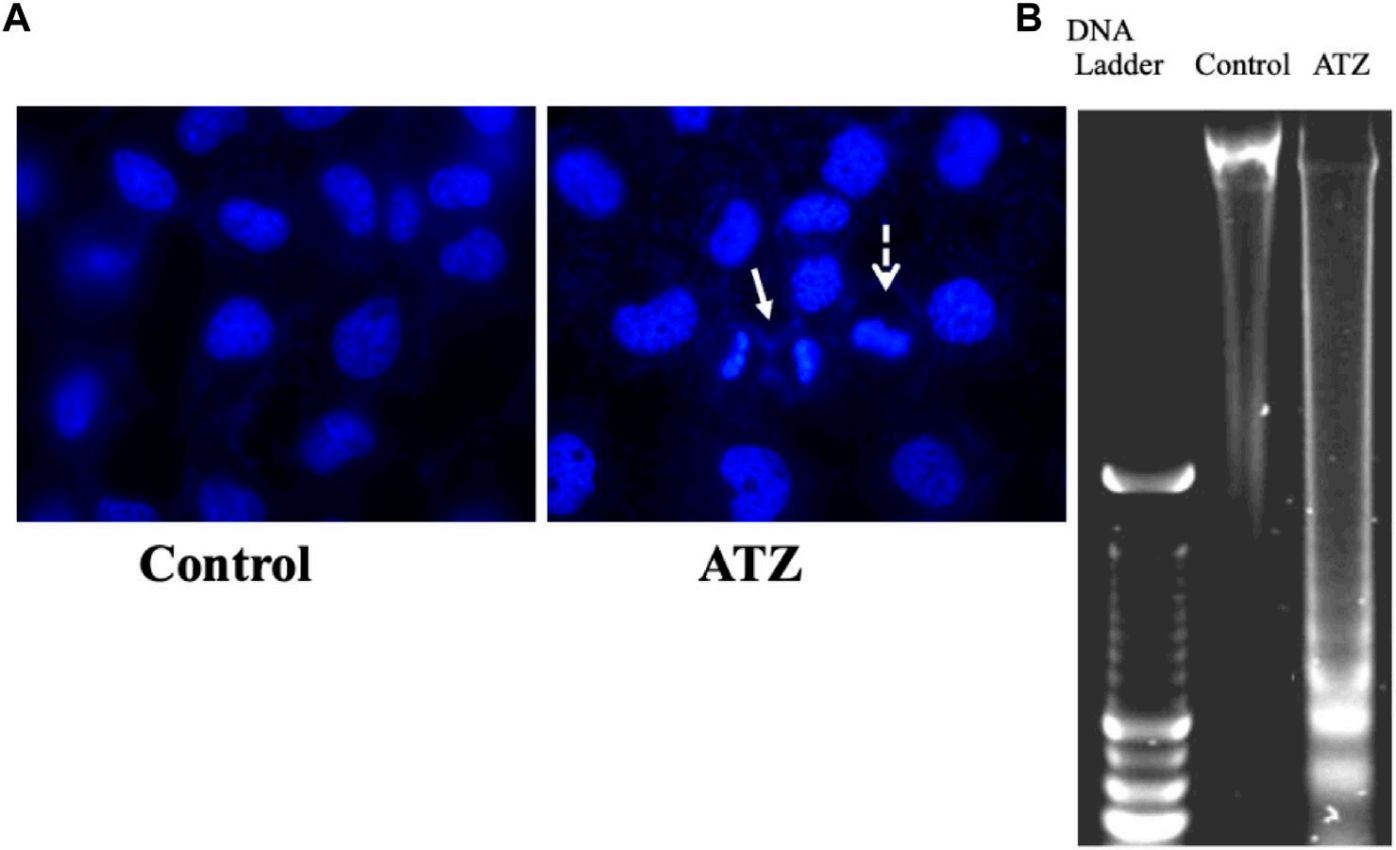
Nuclear changes **(A)** and DNA-fragmentation **(B)** in SH-SY5Y cells exposed to 300 μM of atrazine after 48 h culture period. Source: Abarikwu et al., 2011c.

There are numerous possible course of toxicity that would lead to the suppression of cell growth. One conceivable rationale for the decrease in the cell number could be that ATZ exposure cause DNA damage in cells and ultimately apoptosis. In the research of Olejnik and Colleagues, the detection of the DNA damage was conducted utilizing the alkaline single cell micro electrophoresis assay, a helpful and responsive technique for assessing DNA damage (Ventura et al., 2008). Their results showed that intestinal Caco-2 cells responsiveness to ATZ-induced DNA damage was not remarkably contingent on the growth stage and that Caco-2 cells were not sensitive to the genotoxic effect of ATZ at concentrations of up to 50 μM (Figure 1). To understand the reasons for the diminished cell numbers after exposure of cells to ATZ, propidium iodide staining of DNA accompanied with flow cytometry analysis was used to determine if the ATZ treated cells were adjusted in their successions along the cell cycle (Powell et al., 2011). After exposure to ATZ for 48 h, an aggregation of the S phase cells was observed in the 100, 300 and 500 ppb treated samples (Figure 2) and a decrease in the G1 and the G2/M phases cells in the 300 ppb treated cells for 24 h (Powell et al., 2011). This is the same as the report of Freeman and Rayburn (2006) where CHO cells exposed to ATZ at 200 μM (43,000 ppb) produced a notable aggregation of S phase nuclei. In the study of Powell and colleagues a much smaller concentration of ATZ (100 ppb) was indispensable to initiate an effect. The capacity of ATZ to cause DNA fragmentation and apoptosis in fish cell lines was found to include interference on the mitochondrial membrane potential and production of ROS (Liu et al., 2006). Likewise, the clastogenicity of ATZ in the peripheral lymphocytes of rats was accompanied with escalated expressions of p53 proteins (Cantemir et al., 1997). Experiments developed by our research group also reported that high concentrations of ATZ (300 μM) initiated nuclear changes linked with apoptosis; including fragmentation of nuclei, condensation, DNA laddering (Figure 3), and amplified caspase-3 activity in the human neuroblastoma SH-SY5Y cells. This was associated with alterations in the expressions of caspase-3, caspase-9, p21, p53, Bax and Bcl-2, p21, and and decreased proliferation and growth of cells (Abarikwu et al., 2011c). The diminished expressions of *c-fos* and *c-Jun* in PC12 cells as observed earlier by us was as a consequence of the observed apoptotic cell death as demonstrated by the MTT assay (Abarikwu et al., 2011b). The alterations in the expressions of apoptosis-related genes implicate the role of apoptosis in the observed cell death. Flow cytometric examination established the implication of ROS in the ATZ-induced apoptosis *in vitro*. We conclude here that the increased ROS levels and alterations in the gene expressions of apoptosis-related genes and proteins in experimental models exposed to ATZ indicates the potential of ATZ to induce apoptosis in mammalian models especially at high concentrations.

### 1.3 The cytotoxicity of ATZ. data from *In Vivo* study

The DNA-damaging potentials of ATZ in polychromatic erythrocytes of mice were not evident as measured by the bone marrow micronuclei assay, even at large dose exposures that result in bone marrow suppression and/or death (Kligerman et al., 2000b). Other studies reported that, even extremely high concentrations of the triazines, including ATZ, have only marginal DNA-damaging activity *in vivo* in mouse leukocytes, when the isolated leukocytes were subjected to DNA damage analysis using the alkaline single cell gel electrophoresis assay, one of the most responsive DNA damage protocols available (Tennant et al., 2001). DNA breaks and alkali labile lesions were found in stomach mucosa and kidney, and to a lower degree in the liver, but not in the lungs of rats that were orally administered a single high dose (875 mg/kg b.w) or repeated daily doses (350 mg/kg b.w) of ATZ (Pino et al., 1988) for 5 and 15 days. Interestingly, the DNA damage in the stomach mucosa environment as detected by the DNA alkaline elution technique correlated with the activation of ATZ in the gastic cells (Rajkovic et al., 2011). This observation, couple with the insolubility of ATZ in water and the prolonged contact of ATZ with the stomach mucosa environment, was the reason for the long-lasting DNA-damaging activity of ATZ in gastric cells (Adler, 1980; Rajkovic et al., 2011). High levels of ATZ were reported in the kidneys and liver of mice after single oral exposures to ATZ (5–250 mg/kg b.w) using liquid chromatography/mass spectrometry ((Ross et al., 2009). In rat´s liver, ATZ inhibited the activities of principal enzymes of gluconeogenesis including hexokinase, glucokinase and glycogen synthase resulting in reduced accumulation of glycogen in hepatic tissues, early signs of cytotoxicity and decreased body weight (Gluzczak et al., 2007). This was also detected in the liver of fish (Curic et al., 1999). Atrazine exposure to the Neotropical fish, *Astyanax altiparanae* promoted oxidative stress in their gills, liver and muscles. For example, glutathione *S-*transferase (GST) was decreased and CAT activity was increased in gills. Lipid peroxidation increased in the liver, but endocrine parameters were not altered except for the disruption of the triiodothyronine to thyroxine (T3/T4) ratio, suggesting that in this fish model of ATZ toxicity, oxidative stress variables are better indicators of environmental stress than endocrine disruption. The histological features of the liver included enlarged sinusoid capillaries, vascular congestion and enhanced leukocyte infiltration. The diameter of the hepatocytes and cell size and the hepatocyte nucleus diameter were small. Therefore, authors hypothesized that at low ecologically pertinent and applicable concentrations of ATZ (0.5–10 μg/L), oxidative damage and histological abnormalities in the adult Neotropical fish are better indicators of ATZ toxicity (Destro et al., 2021).

Atrazine was found to increase CAT levels and maintained the expressions of superoxide dismutase (SOD) and GST in the liver of rats. In addition, lipid peroxidation, degeneration of hepatic tissues, activation of heat shock protein-90, escalated expression of connexin mRNA, and genotoxic damage were reported in the liver of the animals. It was concluded that ATZ prompted hepatic oxidative stress that provoked defense mechanisms of the liver, an adaptive strategy essential for maintaining the morpho-physiological integrity of hepatic tissues (Campos-Pereira et al., 2012). The genotoxic potential of ATZ was confirmed by the induced frequency of micronucleated polychromatic erythrocytes that could also be responsible for the elevated lipid peroxidation and cytotoxicity (Campos-Pereira et al., 2012).The role of oxidative stress in ATZ-induced cytotoxicity was further confirmed in the spleen of mice, at doses of ATZ ranging from 100–400 mg/kg b.w after 21 days of daily oral gavage to mice (Gao et al., 2016). The elevated ROS levels and the depletion of GSH concentrations in the serum were all found to occur in a dose-related manner. Additionally, when the splenocytes were removed and tested by comet assay, an increase in DNA comet tail formation was found, confirming the presence of DNA damage. Interestingly, expressions of antioxidant enzymes genes such as heme oxygenase −1 and glutathione peroxidase-1 responded positively in the spleen. It appears that elevated oxidative stress, amongst other factors are implicated in the immunotoxicity effects of ATZ in mammals (Gao et al., 2016). However, the relevance of this hypothesis remains to be clarified, since these concentrations of ATZ are rarely encountered in real-life human scenarios (Lagunas-Basave et al., 2022; Owagboriaye et al., 2022; Li et al., 2023). The cytotoxicity of ATZ at a concentration of 15 μg/L on the erythrocytes of *Lithobates spectabilis* (male frog, native to Mexico) as detected by the micronucleus test, was accompanied with increases in the areas of melanin-containing melanomacrophage centers, and abnormal histological features of the liver along with a rise in the number of membrane with bumpy surfaces, apoptotic, and necrotic erythrocytes (Méndez-Tepepa et al., 2023). Although liver biomarkers that permit for the examination of liver damage were missing in the study, the ATZ effects such as hepatotoxicity and cytotoxicity on the native frog species are relevant indicators of environmental stress especially in countries where ATZ is widely authorized for use in different irrigation systems increasing its potential to contaminate aquatic systems.

**FIGURE 4:**
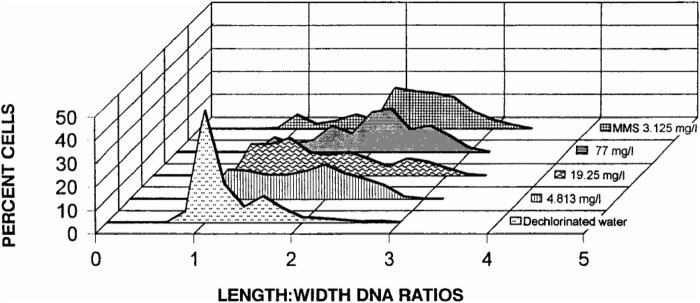
Distribution of DNA damage (based on length-width ratios of DNA patterns) observed at the cellular level in *R. catesbeiana* tadpoles (bullfrog) after exposure for a 24-h period to dechlorinated water. Methyl methanesulfonate (MMS) and selected concentrations of the herbicide AAtrex Nine-O; Source: Clements et al., 1997.

There are still no studies on cell cycle proteins to define the mechanisms of apoptosis and necrosis in frog’s erythrocytes. *Rana catesbeiana* tadpoles (bull frog) exposed to concentrations of AAtrex Nine-O (ATZ as active ingredient) showed significant increases in DNA damage when compared to the control. The percentage of damaged cells was also increased with increasing dose of the herbicide (Figure 4), but the association between DNA damage and the herbicide dose was modest (r = 0.663). Furthermore, no tadpoles lived after 24 h of exposure to 308 mg/L of the herbicide (Table 3), a concentration that is calculated to approximate one-tenth of the oral LD_50_ (3,080 mg/kg) of AAtrex for mice (Clements et al., 1997). It may be assumed that low concentrations of ATZ threaten the survival of organisms that inhabits small bodies of water draining pesticide runoff. The ability of ATZ to also induce genotoxicity *in vivo* concerning the increase in the prevalence of micronuclei and DNA strand breaks was also demonstrated in the erythrocytes of *Carassius auratus* (Cavas, 2011). Cytosol leakage, pyknosis and karyolysis were the common histological features, findings that are indicative of nuclear damage and cell death. The observed cell death agrees with the biochemical data, illustrated by the elevated malondialdehyde (MDA) concentration, tissue degeneration, and over expression of connexions (Campos-Pereira et al., 2012). It was assumed that the cell death occurred by apoptosis, since pyknosis was noticed without the formation of dense bodies and chromatin marginalization, or it could have happened by necrosis because of the notable cell lysis, cytosolic leakage and karyolysis; however, the levels of alanine aminotransferase did not confirm this mechanism. The third assumption on the mechanism of the cell death is autophagy, which was marked by cellular atrophy, nuclear pyknosis and cytoplasmic vacuolation as a consequence of the macroautophagy (Yin et al., 2008). Because a TUNEL reaction in the liver of the rats models (Campos-Pereira et al., 2012) did not confirm cell death, it was thought that the mechanism of cell death induced by ATZ *in vivo* needs to be verified. The glycogen and lipid content in the liver of tadpoles (*Xenopus laevis*) that were exposed to ATZ was unchanged (Zaya et al., 2011). The liver of the tadpole was smaller and those exposed to higher concentration of ATZ (400 μg/L) had larger numbers of activated caspase-3 immuno-positive cells, suggestive of increased rates of apoptosis. The authors believed that the observed changes in body and organs as well as fat body size suggested that ATZ exposure compromised the development of tadpoles (Zaya et al., 2011). Possibly, ATZ decreases the ability of tadpoles to transform and survive the stresses of metamorphosis or diminish their reproductive fitness. The latter is rationale to assume since frogs depends on lipid storage for these molecular events. Histological changes were also found in the liver of ATZ treated zebrafish (*Danio rerio*). ATZ-induced vacuolar degenerations of the liver, biliary hyperplasia and renal tubular necrosis have also been demonstrated in the male Japanese quail, *Coturnix japonica* (Hussain et al., 2011).

**TABLE 3:** Detection of DNA damage in erythrocytes of *R. catesbeiana* tadpoles exposed to AAtrex Nine-O for 24 h (Source: Clements et al., 1997).

Dose (mg/L)	No. of tadpoles	DNA length: width ratio ± SEM^a^	Range: SD ratio
Negative control	11	1.343 ± 0.022	2.99
4.813	7	1.821 ± 0.095	2.38
19.25	6	1.861 ± 0.138	2.52
77	7	2.131 ± 0.087	2.71
308^c^	4		
Positive control (3.125 mg/L MMS)	9	2.265 ± 0.044	3.45

*The recommended application concentrations ranged from 2.5 to 29.3 g/L.

^a^ Ratios based on 25 cells/tadpole.

^b^ All comparisons are relative to the negative control.

^c^ All animals died within 24 h.

MMS, methyl methanesulphonate.

**FIGURE 5:**
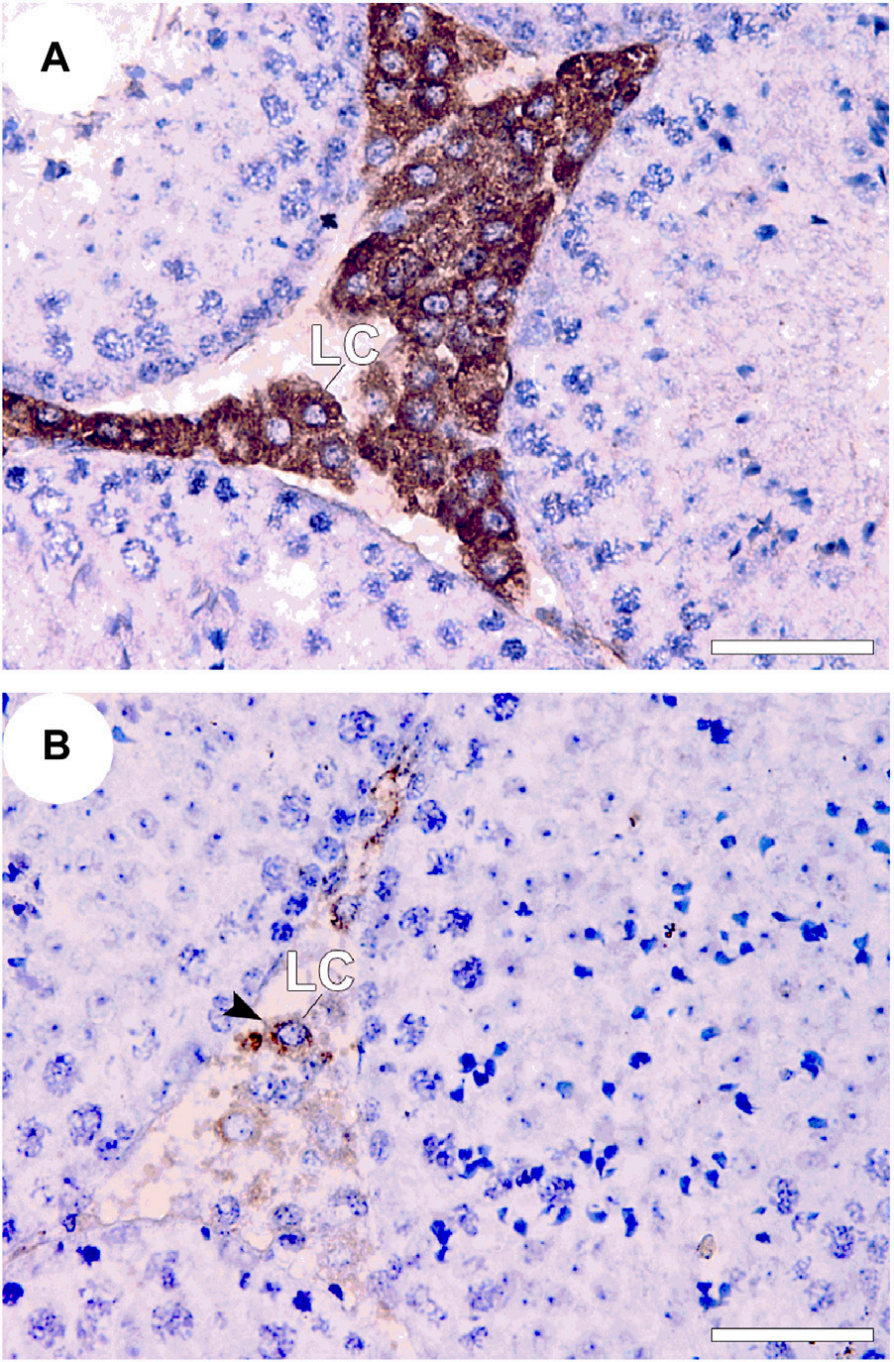
3β-HSD immunostaining in the testes of control and treated BalB/c mice 3 consecutive days after treatment with 50 mg ATZ per kg body wt. The immuno-expression of 3β-HSD was intense in the Leydig cells (LCs) of the control **(A)** and was decreased in the testis of atrazine treated mice **(B)**, Note that 3β-HSD is expressed in almost all LCs in controls, but is only expressed in some LCs in atrazine treated mice, whereas other LCs are less immunopositive for 3β-HSD (arrowhead). Scale bar = 50 μm, Mag 400×). Source: Abarikwu et al., 2021.

To further support the role of apoptosis in ATZ toxicity, low numbers of 3β-hydroxysteroid dehydrogenase positive Leydig cells accompanied with amplified *in situ* cell death fluorescence (Figure 5) and caspase-3 immunoexpression in the testes of mice orally exposed to ATZ (50 mg/kg b.w) for 3 days was demonstrated by us (Abarikwu et al., 2021). The same study also reported elevated immunoexpression for cell cycle gene regulators, including p45, cyclin D2 and E2, suggesting that ATZ bluntly diminish the population of testosterone producing Leydig cells in mice by apoptosis (Abarikwu et al., 2021). It is however possible that metabolites of ATZ are more toxic than ATZ in terms of their endocrine disrupting effects, since diaminochlorotriazine inhibited both serum and testicular testosterone concentrations in mice, unlike ATZ that decreased only testicular testosterone concentrations (Jin et al., 2014).

Another study performed in zebrafish also observed alterations in the expressions of proteins associated with oxidative stress, oncogenesis, lipid metabolism and insulin resistance after ATZ exposures (Yuanxiang et al., 2012). Lipid droplets were found to be accumulated in the liver of rats after 5 months of exposure to ATZ and this was associated with altered mitochondrial morphology in the liver, and additionally in muscle. Since no treatment-associated changes in food or water intake or physical activity were reported throughout the study, it was suggested that the occurrence of insulin resistance as a consequence of ATZ exposure might be related to energy metabolism, and authors suggested that prolonged exposure to ATZ might contribute to the development of insulin resistance and obesity, especially where a high fat diet is fashionable (Lim et al., 2009). Evidence of oxidative stress was also reported in the liver of a fresh water fish Channa Punctatus (Bloch), when the animals were exposed to ATZ at concentrations ranging from 4.24 to 10.6 mg/L in a semi static system for 15 days (Nwani et al., 2010). After completion of ATZ exposure, the antioxidant enzymes: superoxide dismutase, catalase and glutathione reductase responded positively to the enhanced lipid peroxidation status of the fish liver in a concentration-dependent fashion (Nwani et al., 2010). From these data, it can be suggested that the liver is one of the important target organ of ATZ toxicity, but the hepatotoxicity in mammalian models appears to be observed only at concentrations of ATZ not likely encountered in the environment, which may not be true for aquatic animals. However, more studies with environmentally applicable doses of ATZ will furnish additional information on the roles of apoptosis and oxidative stress in the hepatotoxicity of ATZ in human, wildlife and mammalian models.

## 2 Testicular toxicity of atrazine in experimental models

The adverse effect of ATZ on testicular functions has been investigated by few researchers (Table 4). A study developed by Swan et al. (2003) was the first to explore the human reproductive risks that are connected to ATZ exposure. This population-based study demonstrated the link between specific biomarkers of environmental exposures and male reproduction in humans and concluded that the association between pesticides use and diminished semen quality suggested that agrochemicals such as ATZ may have played a part in the lowering of semen quality in fertile men.

**TABLE 4:** Expressions of markers of steroidogenesis in the testis and testicular cells of rats and mice after atrazine exposure as reported in literature.

Parameter	Cell types/tissue	Concentrations/doses	Expressions	References
3β-HSD	Testis	>50 mg/kg	Decreased	Victor-Costa et al. (2010)
3β-HSD	Leydig cells	5–50 μg/mL	Increased	Abarikwu et al. (2012)
AR	Sertoli-germ cells	50 μg/mL	Increased	Abarikwu et al. (2012)
ER-alpha	Sertoli-germ cells	50 μg/mL	No change	Abarikwu et al. (2012)
ER-alpha	Leydig cells	10 μg/mL	Increased	Abarikwu et al. (2011a)
SCF	Sertoli-germ cells	50 μg/mL	Decreased	Abarikwu et al. (2012)
CYP11A1	Leydig cells	5–10 μg/mL	Increased	Abarikwu et al. (2011a)
StAR	Leydig cells	5–10 μg/mL	Increased	Abarikwu et al. (2011a)
LHR	Testis	50–200 mg/kg	Decreased	Pogrmic et al. (2009)
17β-HSD	Testis	50–200 mg/kg	Decreased	Pogrmic et al. (2009)
StAR	Testis	50–200 mg/kg	Decreased	Pogrmic et al. (2009)
CYP17A1	Testis	50–200 mg/kg	Decreased	Pogrmic et al. (2009)
SR-B1	Testis	50–200 mg/kg	Decreased	Pogrmic et al. (2009)
17β-HSD	Leydig cells	20 μM	Increased	Pogrmic et al. (2009)
CYP-17A1	Leydig cells	20 μM	Increased	Pogrmic et al. (2009)
SF-1	Leydig cells	20 μM	Increased	Pogrmic et al. (2009)
StAR	Leydig cells	20 μM	Increased	Pogrmic et al. (2009)
3β-HSD	Testis	50 mg/kg	Decreased	Abarikwu et al. (2021)
ARO	Testis	50 mg/kg	Decreased	Abarikwu et al. (2021)
AR	Testis	50 mg/kg	Increased	Abarikwu et al. (2021)
ER-α	Testis	50 mg/kg	Increased	Abarikwu et al. (2021)
p450scc	Testis	100–200 mg/kg	Decreased	Jin et al. (2013)
p450 17α1	Testis	100–200 mg/kg	Decreased	Jin et al. (2013)
17β-HSD	Testis	100–200 mg/kg	Decreased	Jin et al. (2013)
SR-B1	Testis	200 mg/kg b.w	Decreased	Jin et al. (2014)

3 β-HSD, 3β-Hydroxysteroid steroid dehydrogenase; AR, androgen receptor; ER-alpha = estrogen receptor-alpha; SCF, stem cell factor; CYP11A1 = cytochrome P450 cholesterol side-chain cleavage enzyme (P450scc); StAR, steroidogenic acute regulatory protein; CYP17A1 = cytochrome P450 17α-hydroxysteroid dehydrogenase (P450 17α); SF-1, steroidogenic factor-1; ARO, aromatase; scavenger receptor class B type 1 (SR-B1); 17β-HSD, 17β-Hydroxysteroid dehydrogenases.

**FIGURE 6:**
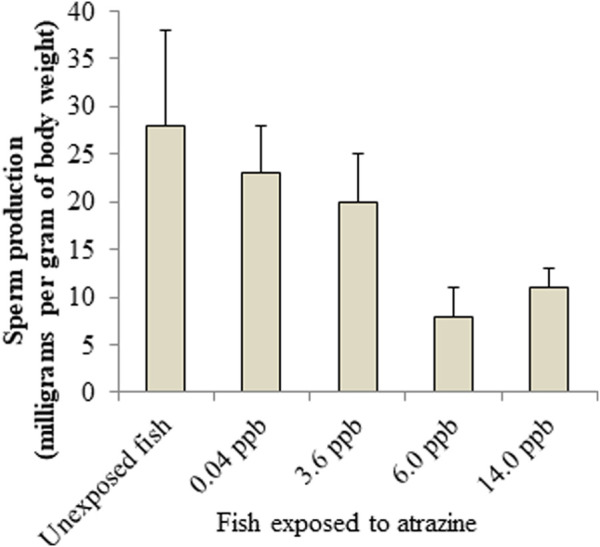
Atrazine decreases sperm production in Salmon. Sources: Moore and Waring, 1998; Cox, 2001.

**FIGURE 7:**
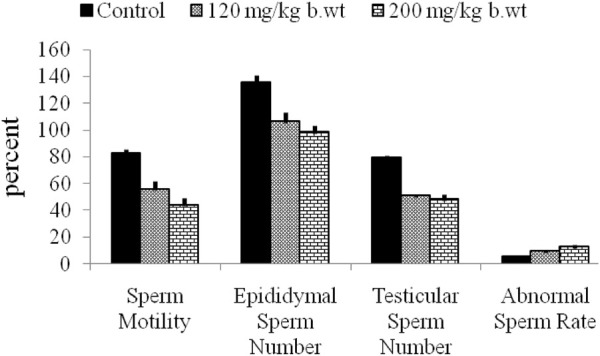
Atrazine decreases sperm quality of rats after 7 days of exposure. Adapted from Abarikwu et al., 2010.

In animal studies, it was shown that histopathological features in the testis and epididymis of ATZ treated mice revealed cells that were disorganized and clusters of cells aggregated with spermatocytes (Abarikwu et al., 2021). The electron microscopy of the testicular tissue of rats displayed separately vacuolated cytoplasm, diminished collagen fibre and presence of Sertoli cells with degenerated cytoplasm (Kniewald et al., 2000). Additionally, Leydig cells were of eccentric shape with dissimilar structure and the cisternae of rough endoplasmic reticulum were more noticeable and softly expanded (Kniewald et al., 2000). The Leydig cells cytoplasmic protuberances were minuscule and the intercellular space was sizeable, displaying larger bulk of collagen fibers. Furthermore, the association between Leydig cells and macrophages were also floppier, and macrophages showed scanty lysosomes, patchy nuclei, commonly with profound folds and accentuated nucleoli (Victor-Costa et al., 2010). The altered testis morphology was evidenced by few atrophic seminiferous tubules and their dilation at ATZ doses of 200 mg/kg and 300 mg/kg for 15 and 7 days respectively, and some enlarged tubules appeared that are uneven in shape. Considering these irregular appearances of the tubules, authors did not measure the alterations in the testes. Conversely, the testes of rats treated with ATZ at 200 mg/kg for 40 days were marked by atrophy and substantial reduction in the lumen of the seminiferous tubules. Interestingly, most of the seminiferous tubules in this group of rats were Sertoli cells only. Large multinucleated bodies and numerous apoptotic cells were also common features in the seminiferous tubules (Victor-Costa et al., 2010). Thin layers of cells close to basement membrane with expanded intertubular space in rats exposed to 50 mg/kg body weight ATZ for 60 days were also confirmed by us (Ndufeiya-Kumasi et al., 2022), and when the same dose was applied in BalB/c mice for 3 days, the gonads displayed diminished numbers of germ cells in tubules with large apoptotic cells near the lumen as well as few numbers of Leydig cells (LCs) in the intertubular areas (Abarikwu et al., 2021), supporting the testicular toxicity of ATZ in mammalian animal models. The testicular lesions induced by ATZ were also accompanied with decreased germ cell numbers in amphibians, teleost fish and reptiles (Figure 6), a feature that appears to be congruous across vertebrate classes (Hayes et al., 2011). In an earlier report by Hayes and colleagues (2003), ATZ demasculinizes and feminizes the male gonads of vertebrates by decreasing androgen levels and inducing estrogen synthesis. This feature has been successfully demonstrated in fish, amphibians and reptiles, and may represent the probable mechanisms to explain these effects. It was also found that ATZ decreased testicular testosterone level in male rats and mice (Friedmann, 2002; Abarikwu et al., 2021) and this was accompanied with poor semen quality in humans (Swan et al., 2003). This finding agrees with the poor sperm parameters including, membrane integrity, mitochondrial functionality, decrease acrosome integrity of sperms obtained in the mice, *Calomys laucha* after 21 days exposure of the animal to low dosages of ATZ (Saalfeld et al., 2018). In addition to the decrease in the levels of serum and intratesticular testosterone, following ATZ exposure in mice, we also detected notable decline in the levels of pituitary hormones in our recent study (Ikeji et al., 2023). This finding suggests that ATZ may induce dysfunction in hypothalamus orchestrating an adverse reaction in the secretion of gonadotropins and testosterone, thus perturbing the hypothalamic-pituitary–gonadal axis which plays pivotal role in male reproductive functions. We also demonstrated that sperms obtained from the cauda epididymides of rats exposed to ATZ up to 14-days were of poor quality as reflected in the observed low motility, count and high abnormality rate (Figure 7) (Abarikwu et al., 2010) as well as decreased daily sperm production, 7 days post ATZ exposure (Abarikwu et al., 2012). The testis of ATZ treated Japanese quail (*Coturnix japonica*) was diminished in size and the seminiferous tubules revealed reduced numbers of spermatocytes, spermatids with necrotic nuclei and decreased numbers or lack of spermatozoa (Hussain et al., 2011), similar to the observation found in rats for gonadal weight effects ((Abarikwu et al., 2010) and in mice for effect on germ cells (Abarikwu et al., 2021; 2022).

The relevance of the mRNA expressions of steroidogenesis genes: steroidogenic acute regulatory protein (STAR), cytochrome P450 (CYP) 11A1, 3β-hydroxysteroid dehydrogenase (3β-HSD), estrogen receptor-alpha (ER-α) and androgen receptor (AR) in interstitial Leydig cells (ILCs) as a gauge of ATZ induced testicular toxicity in rats ILCs after short-term exposure was reported by us (Abarikwu et al., 2011a). At the level of *in vivo,* immunoexpression of LCs populations from mice sub-acutely exposed to ATZ that were immunopositive for 3β-HSD were dramatically decreased (Figure 5) whereas those of ER-α, aromatase and AR were increased (Abarikwu et al., 2021), pointing to LCs as the primary cell that is sensitive to ATZ exposure in adult mice. The fact that most of these cells were TUNEL positive and were immunopositive for caspase-3 confirmed this assertion (Abarikwu et al., 2021). Furthermore, regarding the capacity for ATZ to induce steroidogenesis genes expressions and activities of CYP17A1 and 17β-HSD, cAMP builds-up, and androgen production were found to be transient resulting to the facilitated androgenesis (Pogrmic-Majkic et al., 2010). Conclusively, Pogrmic-Majkic et al. (2010) demonstrated from their study that *in vivo* exposure to ATZ compromises LCs steroidogenesis by down-regulating steroidogenesis gene expression associated with diminished androgenesis. The capacity of ATZ to change the expression of genes related to spermatogenesis steroidogenesis was also detected in Sertoli-germ cells co-culture and was accompanied with decreased testicular sperm production *in vivo* (Abarikwu et al., 2012). Atrazine at doses higher than 50 mg/kg decreased body weight, elevated adrenal weight and transiently elevated testis weight, accompanied with testis atrophy. The decrease in testosterone was associated with enhanced estradiol concentrations (Victor-Costa et al., 2010) and changes the transcription of the key genes in the testosterone synthetic pathway (Jin et al., 2014). The study of Victor-Costa and colleagues was the first time 3β-HSD protein was observed to decrease in the testes even when it remained unchanged in the adrenal. This is a very interesting finding, because it suggests that 3β-HSD inhibition may represent a probable route through which ATZ impairs androgen production in the testes resulting to the distortion of spermatogenesis in adult mammalian animal models. Our recent findings on the low populations of 3β-HSD^+^ LCs at the inter-tubular spaces of mice testes treated sub-acutely to ATZ for 3 days confirms that the impaired testicular androgenesis were due to decreased expression of 3β-HSD^+^ LC (Abarikwu et al., 2021), and supports the hypothesis of 3β-HSD inhibition by ATZ in rodents as opposed to the aromatase induction hypothesis in lower animals (Hayes et al., 2011; Abarikwu et al., 2021), as a plausible mechanism for the decline of testicular androgen production in rodents. Another major concern has been if ATZ enhances estrogen synthesis, perhaps by intensifying aromatase gene expression and activity. To verify this, Tinfo et al. (2011), compared the effect of ATZ ón primary cultures of granulose cells and H295R adrenal cortical carcinoma cell lines, and reported elevated estradiol production and aromatase activity in granulose cell cultures, but not in the H295R cells, that were characterised with escalated estradiol and estrone production only. Apparently, the enhanced progesterone production in both cell types demonstrates a broader effect of ATZ on steroid production (Tinfo et al., 2011).

To understand the importance of the antioxidant protective network in the testis and epididymis of mammalian animal models, we found that the antioxidant defense set-up in the gonads, e.g., testis and epididymis was decreased, a scenario that is synonymous to the manifestation of oxidative stress (Abarikwu et al., 2010). Our research group reported that glutathione (GSH) concentration and GST activities were increased in rats treated with ATZ at 200 mg/kg b.w., whereas lipid peroxidation levels were unaffected in the testis 7-days post-exposure. When the treatment regimen was elongated to 16 days, GSH concentration remained unaffected whereas malondialdehyde concentration was escalated both inthe testis and epididymis. This correlated to the decrease in GST and superoxide dismutase (SOD) enzymatic activities. Catalase (CAT) activities were unaltered in the testis and then diminished in the epididymis. During the 7-day exposure regimen, the variables of sperm quality including epididymal and testicular sperm numbers and sperm morphology were altered even though there was no evident of oxidative stress (Abarikwu et al., 2010). In addition, the histology of the testis and epididymis were normal. Since the spermato-toxicity occurred earlier than when oxidative stress was manifested, authors inferred that oxidative stress in the testis and epididymis of rats after ATZ exposure might not be responsible for the impaired sperm quality. Doses of ATZ and metabolites of ATZ similar to doses reported in the above studies have also shown to induce oxidative stress in different experimental model systems (Jin et al., 2014), supporting the oxidative stress hypothesis in the toxicity of ATZ. These effects seem to occur at doses higher than environmental levels and unlikely to occur at lower doses ((Saalfeld et al., 2018), and thus, ATZ effect on the variables of oxidative stress in the testes of mammalian animal models may not exceed a level of concern for humans (Saalfeld et al., 2018). However, in studies where ATZ at much lower doses (10 mg/kg b.w) was found incapable of altering lipid peroxidation status in sperm isolates, the sperm quality markers such as progressive sperm motility were lowered even at doses lower than environmental levels, justifying the claims that sperms in epididymis are responsive to ATZ toxicity than oxidative stress variables in the testis (Abarikwu et al., 2010). To support this assertion, Jestadi et al. (2014) did not observe any testicular toxic effects in rats treated with low doses of ATZ. However, the ´´no observed effect´´ dose of ATZ (0.5 mg/kg b.w) set by the Australian government was demonstrated to alter the sperm quality profiles of weaning mice at 12 weeks of age (Cook et al., 2018). Thus, the low dose effects of ATZ on the metabolic and reproductive features of mammalian male animal models are influence by the age at which the exposure to ATZ occurs.

The capacity of ATZ to alter electron transport and oxidative stress in the mitochondria of *Drosophila melanogaster* has also been reported (Thornton et al., 2010). In a panel of research designed to determine the possible effects of ATZ on male amphibians *in vivo*, gonadal aromatase activity in ATZ-treated animals was found to be comparable to those of the control animals (Coady et al., 2004; Hecker et al., 2004). The explanation for the contrary results was attributed to one of two factors: 1) ATZ does not affect aromatase activity in the examined amphibian species, or 2) that because of the inadequate enzyme activities in the gonads, it was not technically workable to analyse precise variations in aromatase with the applied test model. In another study with *Xenopus laevis*, aromatase enzyme activity and gene expression were comparable to the control, and the tested ATZ concentration was found not to interfere with steroidogenesis because of the unaffected aromatase action (Hecker et al., 2005). Thus, there remain discrepancies on the hormonal effects of ATZ in experimental models, and only at high concentration was ATZ found to alter plasma testosterone homeostasis. Future studies with environmentally relevant doses of ATZ targeting more on a general mode of toxic action as pertaining to their effects on spermatogenesis and steroidogenesis are therefore necessary.

## 3 Chemopreventive intervention with selected antioxidants

Several pharmacological agents including melatonin, vitamins, quercetin, kolaviron, selenium, tannic acid have been used to reduce ATZ-induced toxicity regarding oxidative damage and apoptosis. These molecules substantially decreased the *in vivo* and *in vitro* toxic actions of ATZ, presumably, because of their antioxidative properties. Melatonin, a principal secretory product from the pineal gland, has been demonstrated to have an effective antioxidant and free radical scavenger properties (Buyukokuroglu et al., 2008). The *in vivo* administration of ATZ in rats inhibited glucose-6-phosphate dehydrogenase activity, ATPases (e.g., Na^+^/K^+^-ATPase, Mg^2+^-ATPase, and Ca^2+^-ATPase), and diminished protein, total lipids, cholesterol, and phospholipid concentrations in erythrocyte membrane. Scanning electron microscopic examination showed structural modifications in the erythrocytes of ATZ treated rats. However, melatonin supplementation adjusted the ATZ-induced lipid peroxidation level and the variations in total lipids, ATPases activities as well as GSH concentration and antioxidant enzymes, confirming the antioxidant protective actions of melatonin against ATZ-induced oxidative impairment in rat´s erythrocytes (Bhatti and Sidhu, 2011). Additionally, melatonin upregulated the expressions of E2F-1 and PUMA and diminished Bax expression in an ATZ model of p53 independent mitochondrial apoptosis (Sharma et al., 2014). The endoplasmic reticulum (ER) stress as a result of the enlarged expression of ATF-6α, spliced XBP-1, CREB-2 and GADD153 was also attenuated by melatonin. This molecular event that was also accompanied with the expression LC3B-II and p62 and diminished BECN-1 signals were also attenuated by melatonin. The cytoprotective role of melatonin in an ATZ model of p53 independent mitochondria-mediated apoptosis, autophagy and ER stress was thus established by the Sharma and colleagues (2014). Tannic acid, a glucosyl chemical in gallnuts, has been demonstrated to antagonize ATZ (3 ppb)-induced Grass carp hepatocytes cytotoxicity (Gao et al., 2022)**.** The application of both flow cytometry and dual acridine orange/ethidium bromide fluorescent staining demonstrated a higher ratio of apoptosis and necrosis in the hepatocytes. Additionally, the oxidative stress-related indicators, including ROS and MDA levels that were elevated, and the downregulated anti-oxidative system found after ATZ exposure, were alleviated by tannic acid. The mechanism for the cytotoxicity of ATZ in fish hepatocytes as proposed by Gao and colleagues involves the binding of tumor necrosis factor α (TNF-α) to TNF receptor 1 (TNFR 1) resulting in the upregulated expressions of the markers of apoptosis and necrosis: TRADD, FADD, Caspase-3, p53, RIP1, RIP3 and MLKL, whereas tannic acid abrogated the-induced apoptosis, necrosis and immunotoxicity through a ROS-responsive TNF-α/TNFR 1 pathway (Gao et al., 2022). In an ATZ model of rat hepatic injury, L-carnitine at 100 mg/kg b.w demonstrated a strong potent antioxidant, anti-inflammatory and antiapoptotic properties that had ameliorative effect against ATZ-induced hepatotoxicity (Rashad et al., 2023). In this study, 400 mg ATZ/kg b.w administered daily for 2 weeks induces adverse functional alterations and morphological changes in the rats that were accompanied with increased activities of liver enzymes and oxidative stress, modified expression of apoptotic and antiapoptotic genes, degenerative changes in the liver, and robust immunoreactivity to glial fibrillary acidic protein (GFAP), supporting previous findings on the oxidato-inflammatory and apoptotic mechanisms of ATZ in mammalian models (Campos-Pereira et al., 2012). In a similar study, vitamin E protected against ATZ-prompted lipid peroxidation in the liver and reversed the GSH level and the activity of glucose-6-phosophate dehydrogenase that was found to decline after ATZ treatment. It was deduced that ATZ promoted oxidative stress through lipid peroxidation, and that vitamin E supplementation attenuated these effects, demonstrating it as a possible antioxidant protective molecule against oxidative stress initiated by ATZ (Singh et al., 2011). Administration of selenium to rats by oral gavage did not counteract ATZ-induced impairments of sperm quality and had no beneficial protective effects against the alterations in the biochemical variables caused by ATZ the testis and epididymis. However, selenium strongly protected against the biochemical changes initiated by ATZ in the liver. Thus, selenium was found to be valuable in reversing hepatic injury but not the reprotoxicity of ATZ (Adesiyan et al., 2011). We attributed this observed pattern of selenium protective effect in ATZ model of hepatotoxic and testicular injury in rats to the disparate redistribution of selenium in the target tissues (Adesiyan et al., 2011). One limitation of this study was that it did not measure the selenium contents both in hepatic and gonadal tissues. In a recently published article, the protective effects of zinc oxide nanoparticles and vitamin C against ATZ-induced hepatotoxicity was explored in rats. After exposing the animals to a single repeated oral dose of ATZ (300 mg/kg b.w) for 21 days, ATZ was also found to elevate liver oxidative stress by prompting the formation of MDA at higher level and decreasing GSH concentration. These biochemical alterations were found to induce inflammation associated with apoptosis through up-regulating Bax, nuclear factor-kappa B, TNF-α and minimising Bcl-2 gene expressions in the hepatic tissues. Light microscopy of liver sections was typified with vacuolated and degenerated hepatocytes surrounding dilated and congested blood sinusoids and central veins (Mohammed et al., 2023). Additionally, the serum samples obtained from the animals were characterised with higher level of aspartate aminotransferase and alanine aminotransferase enzyme activity, and diminished albumin and globulins concentrations confirming liver toxicity. Interestingly, zinc oxide nanoparticles (10 mg/kg b.w) or vitamin C (200 mg/kg b.w) pre-treatment through the oral route for 30 days could dampen the oxidative stress, inflammation, and apoptosis orchestrated by ATZ, suggesting that zinc oxide nanoparticles and vitamin C supplementations can effectively protect the liver from ATZ-hepatotoxicity (Mohammed et al., 2023). Because, the dose 300 mg/kg b.w of ATZ is in the same range of doses as in other *in vivo* studies of ATZ model of experimental hepatotoxicity (Santa Maria et al., 1987; Campos-Pereira et al., 2012; Rashad et al., 2023) or higher than those used in other ATZ mammalian models of experimental toxicology (Liu et al., 2014; Liu et al., 2021; Khozimy et al., 2022), the hepatic injury observed in most of these studies above is of limited clinical relevance. Therefore, it is only at experimental doses, and not at relevant environmental concentrations has ATZ-induced hepatotoxicity been established for mammalian animals. However, one study (Jestadi et al., 2014), have demonstrated hepatotoxicity associated with elevated serum aspartate aminotransferase and alanine aminotransferase activity in animals exposed to ATZ at doses of 300 μg/kg b.w, which is more than 1,000 times lower than was used in the above studies (Campos-Pereira et al., 2012; Mohammed et al., 2023; Rashad et al., 2023). Thus, the proposal that ATZ effects, in mammalian animals may be influenced by differences in methodologies, sources and purity of chemical, solvents, and statistical analyses (Kligerman et al., 2000b), are important concepts to consider in studies focusing on ATZ-induced liver injury in animal models. But, it is also interesting to appreciate that experimental model that is focused on the antioxidant actions of the chemical interventional agent and high doses of the chemical toxicants are important for determining a mechanistic effect of the chemical toxicant (Powell et al., 2011).

Kolaviron, a bioactive bioflavonoid extracted from bitter kola was also found to protect cultured ILCs from ATZ-induced toxicity. Our study with ILCs demonstrated that kolaviron improved the LCs viability and diminished MDA and ROS concentrations. Additional investigations showed a decrease in glutathione peroxidase (GSH-Px), glutathione reductase (GR), GST and escalation of superoxide dismutase 1 (SOD-1) and superoxide dismutase 2 (SOD-2) as measured by mRNA expression. Furthermore, the changes in the mRNA transcript copy numbers of steroidogenesis genes: StAR, CYP11A1, and 3β-HSD prompted by ATZ treatment were reversed to control values, confirming that kolaviron protected ILCs against the toxicity of ATZ. This was speculated to be throughthe suppression of ROS and MDA levels and accompanied by the normalization of mRNA expression of the tested antioxidant and steroidogenesis genes (Abarikwu et al., 2012). This is very interesting considering that a principal defensive strategy against oxidative stress in tissues and cells is the stimulation of the mRNA expression of antioxidant genes. Similarly, our study with quercetin, a flavonol with potent antioxidant properties, showed that quercetin blunted the diminished cell viability and germ cell depletion, and stopped lactate dehydrogenase release caused by ATZ, implying that testicular cell toxicity promoted by ATZ can be arrested by quercetin (Abarikwu et al., 2012) just like kolaviron. Experimental models and designs that combined both kolaviron and quercetin would be important at determining which of them will show better cytoprotective effects in ATZ model of tissue injury related to male reproduction. Because the expressions of cyclooxygenase-2, GATA-4, NF-κB, stem cell factor and androgen receptor in Sertoli-germ cells treated with ATZ were strongly recovered to levels comparable to the control by quercetin, suggested that quercetin might have diminished the capacity of ATZ to initiate spermatogenesis disturbance. The shedding of germ cells from Sertoli cell after ATZ exposure to cultures of Sertoli-germ cells which were inhibited by quercetin could be explained by the fact that quercetin preserved the associations between Sertoli and germ cells, thereby facilitating spermatogenesis (Abarikwu et al., 2012). Quercetin is therefore an important cytoprotective agent against impaired spermatogenesis by environmental factors, such as the triazines.

In a recent study, we confirmed *in vivo* that quercetin at lower doses could improve the histological features of the rat´s testes after treated with ATZ. The tubules with single layers of germ cells including the stem cell spermatogonia next to the basement membrane and enlarged interstitial space after ATZ exposure were seen to have been re-populated with germ cells at different maturity stages in the epithelium over a time course of 60 days after treatment with both curcumin and quercetin (Ndufeiya-Kumasi et al., 2022). Although, curcumin was better than quercetin in protecting the testis from ATZ gonadal toxicity, their combined -treatment improved quercetin protective effects against ATZ-induced testicular injury. It seems plausible because curcumin has a preferable ability compare to quercetin to pass through the blood-testes barrier and on co-administration could promote the passing of quercetin through tissue barriers so as to enhance their protective effect against ATZ-induced injury to the gonads (Sharma et al., 2018; Abdelhamid et al., 2020; Ndufeiya-Kumasi et al., 2022). Additionally, plant extractives like fluted pumpkin seeds that have substantial bioactive substances such as polyphenols that have numerous biological and pharmacological effects on the testes was found to be protective against testicular injury-induced by ATZ in rats by recovery of the sperm quality, biometric data and re-balancing of the MDA and GSH testicular status to the level seen in the control values as well as the activity of testicular lactate dehydrogenase and γ-glutamyl transpeptidase, important enzymes involved in testicular germ cell energetics and epididymal oxidative stress control respectively (Abarikwu et al., 2022). Aside their effect on male reproduction, kolaviron was found to attenuate enhanced ROS generation, cell death and diminished the human neuroblastoma cell line (SHY-SY5Y) proliferation treated with ATZ. Molecular features that characterize nuclear changes due to apoptosis such as DNA laddering, nuclear fragmentation and condensation and escalated caspase-3 activity prompted by ATZ treatment were strongly blunted by kolaviron, including the variations in p53, Bax, Bcl-2, p21, caspase-3 and caspase-9 expressions (Abarikwu et al., 2011c). Similar observations were made with the rat pheochromocytoma (PC12) cell line (Abarikwu et al., 2011b) and confirmed the medicinal prospects of kolaviron in neuronal model of ATZ-induced apoptotic cell death. Myricetin, a flavonoid abundantly found in red wines and grapes, displayed protective effect on testicular injury caused by ATZ, increased levels of the pituitary hormones and recovered normal testosterone production and circulation in both serum and interstitial fluid (Ikeji et al., 2023). Also, myricetin attenuated ATZ-induced diminution in antioxidant enzyme activities and markers of oxidative stress, including the histological aberrations in the epididymis, hypothalamus and testes of mice.

## 4 Molecular insights on the role of apoptosis and oxidative stress in the cytotoxicity of ATZ

**FIGURE 8:**
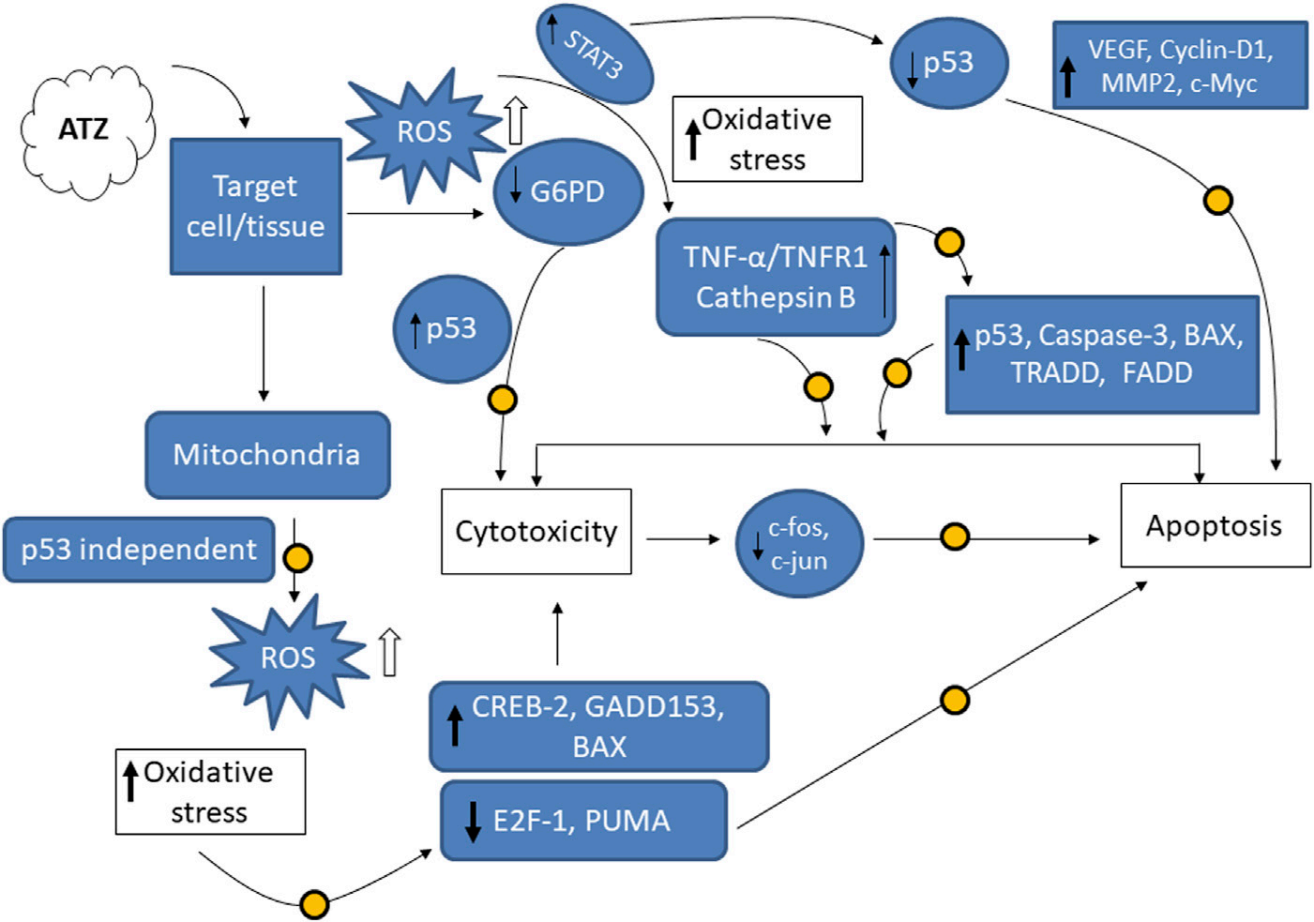
Probable graphical representations of the molecular mechanisms of ATZ-induced redox imbalance, cytotoxicity and apoptosis, as well as possible sites of actions of chemopreventives. The probable interventional target sites for the chemopreventives are illustrated as shaded circles (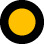).

The probable mechanisms contributing to ATZ–induced cytotoxicity, apoptosis and oxidative stress have been summarized in Figure 8. The binding of ATZ to target cells mitochondrial redox systems may cause mitochondrial dysfunction (Galbiati et al., 2021) and activate ROS- induced oxidative stress systems, followed by cytotoxicity, DNA damage and apoptosis. The initial response to ATZ exposure would involve the inactivation of glucose 6 phosphate dehydrogenase activity/expression, and subsequently, the depletion of intracellular GSH pools (Lioi et al., 1998). If the exposure to ATZ is sustained, the resulting redox-imbalance activates p53 expressions directly, leading to cytotoxic responses in lymphocytes (Lioi et al., 1998), or through a ROS-responsive TNF-α/TNFR 1 pathway, and together with increased cathepsin activity could explain the increased caspase-3 mediated apoptosis (Gao et al., 2022). The cytotoxic response that accompanied decreased c-Fos and c-Jun expressions may result in increased apoptosis (Abarikwu et al., 2011b). Oxidative stress may also then activate, as a defense mechanism, the STAT3 transcription factor (Ng et al., 2014), which in turn downregulates p53 transcription (Tian et al., 2018). The latter, together with increased ROS generation and expressions of VEGF, cyclins (cyclin-D1), MMP2 and C-myc, explain increased cell apoptosis (Tian et al., 2018). The p53-independent mitochondria cytotoxicity pathway activates a ROS responsive upregulation of BAX (Bcl-2 Associated X-protein) expression which together with GADD153 and CREB-2 dampen the expressions of E2F-1 and PUMA to drive apoptosis and cytotoxic damage (Nakano and Vousden, 2001; Sharma et al., 2014). However, the experimental settings, such as the concentration, duration and the age at which exposure to ATZ occurs, may influence differently the above-described mechanisms, possibly explaining the contrasting effects of ATZ in different mammalian model systems (Kligerman et al., 2000b; Galbiati et al., 2021).

## 5 Conclusion

Although ATZ may induce cytotoxicity, oxidative stress, apoptosis and genotoxicity in *in vivo* and *in vitro* experimental models, the majority of the doses of ATZ required to induce these changes in most of the studies reported in literature are unlikely to be found in the environment, thus the environmental levels of ATZ may not exceed a level of concern for humans and wildlife. However, the contributions of oxidative stress and apoptosis in the cytotoxicity of ATZ have been demonstrated in many experimental model systems. But, it is pertinent to consider that, as humans and wildlife may be continuously exposed to minuscule concentrations of ATZ, more studies with environmentally relevant doses of ATZ will come up with further ideas and assumptions on the mechanism of testicular-, neuronal- and hepato-toxicity caused by ATZ. Furthermore, the actions of ATZ on redox status of cells/animals are important interventional target sites at the mechanistic level, since the induced oxidative stress, cytotoxicity, apoptosis and genotoxicity observed in ATZ-exposed experimental models were blunted by antioxidants: such as vitamins (vitamin E and C and antioxidant bioactive flavonoids such as quercetin, myricetin, curcumin and kolaviron. Finally, 3β-HSD inhibition represents a plausible alternative mechanism by which ATZ affects gonadal androgen secretion and production resulting to changes in spermatogenesis in adult mammalian animal models, which might be different from the aromatase hypothesis that have been established for lower animals.
